# Need dissatisfaction and its consequences on support for anti-pandemic behaviors in China: The mediation of attribution and local government satisfaction, and the moderation of social class

**DOI:** 10.3389/fpsyg.2022.1040518

**Published:** 2022-12-14

**Authors:** Yan Zhang, Junxiu Wang

**Affiliations:** ^1^Institute of Sociology, Chinese Academy of Social Sciences, Beijing, China; ^2^School of Mental Health, Wenzhou Medical University, Zhejiang, China; ^3^School of Psychology, Inner Mongolia Normal University, Hohhot, China

**Keywords:** need satisfaction, attribution theory, social identity theory, social class, COVID-19, government satisfaction

## Abstract

**Introduction:**

The COVID-19 pandemic has greatly impacted the global economy, resulting in a substantial increase in inequality. There is a need to understand need dissatisfaction in this context, its group differences, and its consequences on support for anti-pandemic behaviors.

**Methods:**

Using data from a survey round of the Chinese Social Mentality Survey from 21 April to 26 May 2022, 6,022 participants aged between 18 and 70 years (*M* = 32.27; *SD* = 8.74; men = 46.76%) from 29 provinces of Mainland China were included in the study.

**Results:**

1) Need dissatisfaction was negatively related with support for anti-pandemic behaviors and was completely mediated by attribution and local government satisfaction. 2) Internal/external attribution acted as a double-edged sword: they were negatively/positively related with support for anti-pandemic behaviors, while they became positively/negatively related with support for anti-pandemic behaviors *via* the mediation of local government satisfaction. 3) People who were unemployed and in the subjectively middle class reported higher need dissatisfaction and less support for anti-pandemic behaviors compared to their counterparts. 4) Social class moderated the relationship between need dissatisfaction and internal attribution: when needs were dissatisfied, participants with higher income and subjective social class tended to attribute more internally.

**Discussion:**

This study contributes to the attribution theory and social identity theory in the context of major global public health events and provides practical implications for promoting behavioral compliance in the context of COVID-19. In particular, facilitating a positive interaction between the public and local governments may be helpful to create a shared identity and, ultimately, prevent and control the pandemic together.

## Introduction

The COVID-19 pandemic has lasted for more than 2 years and increased world inequality. It has been estimated that by the end of 2022, at least 75 million more people will have been pushed into poverty (living on <US$1.90/day) than was expected before the pandemic (Sidik, 2022). The World Bank revealed that although people at all income levels lost money, the highest earners regained more than half of their losses between 2020 and 2021, whereas the lowest earners did not recover their losses compared with expected increases in earnings (Sidik, 2022).

Deaton (2021) found that when countries were weighted by population, international income inequality increased, not because the poorest countries diverged from the richest countries but because China had positive economic growth, pulling it away from poor countries. As one of the few countries that adhered to strict pandemic prevention and control policies, China is effective in tackling COVID-19 variants, and the domestic pandemic situation remains stable (China Daily, 2022a). However, in April 2022, when global COVID-19 cases topped 500 million, with more than six million deaths, China also encountered the largest COVID-19 spread since 2019. Shanghai was locked down for the first time, and its daily confirmed cases exceeded 10,000. Doubt about the sustainability of these policies started to emerge because of the highly infectious nature of the Omicron variant (China Daily, 2022a). China’s economic growth is slowing, and domestic travel during a weeklong holiday fell below 2021 levels, as a cluster of new cases worried tourists; retail sales have proven fitful, recovering, and ebbing with waves of the virus (Wang, 2021). The subsistence needs of people demand meeting and satisfying. Understanding the consequences of need dissatisfaction on attitudes toward anti-pandemic behaviors in China is thus important to understand why some people adhere to certain pandemic prevention and control policies while others do not, as well as how to ensure the compliance with anti-pandemic interventions during this particular time.

This study aimed to explore the relationship of need dissatisfaction on support for anti-pandemic behaviors. By integrating an individual perspective and a social perspective together based on the Attribution Theory and Social Identity Theory, the study also explored the mechanism between need dissatisfaction and support for anti-pandemic behaviors through the mediation of attribution and local government satisfaction. Finally, the study attempted to study COVID-19 inequality subjectively by exploring whether people in lower classes reported more need dissatisfaction and less support for anti-pandemic behaviors, as well as the moderation role of social class.

### The relationship between need dissatisfaction and support for anti-pandemic behaviors

Needs have been conceptualized in various ways in different sub-disciplines of psychology and at distinct points in time, such as Maslow’s hierarchy of needs, and Deci and Ryan’s basic psychological needs (Maslow, 1964; Ryan and Deci, 2000; Williams, 2009). However, those frameworks of need do not consider the above special unmet needs that emerged because of the pandemic and policies related to it. Our study did not intend to explore psychological need satisfaction but real-life need dissatisfaction during the pandemic.

Need dissatisfaction refers to the feeling and evaluation of the extent to which needs are not met as expected; yet it is not the opposite of need satisfaction (Neubauer and Voss, 2018). One of the differences between the two concepts is that need dissatisfaction predicts motivation to pursue the dissatisfied need, but need satisfaction does not (Neubauer and Voss, 2018). During the COVID-19 pandemic, people have not only been exposed to the risk of being infected by the virus, but also of not being able to satisfy their needs (China Daily, 2022b), since physical distancing interventions that were essential to flatten the epidemic curve and reduce the disease burden (Chung and Chan, 2021) may have caused job losses, business going bankrupt, and economic insecurity. For example, it has been estimated that the catering industry lost 500 billion yuan in 7 days of lockdown in 2020 (Ren, 2020). Other industries, such as travel, hotels, agriculture, and livestock, have also been affected.

Researchers have found that public compliance with anti-pandemic behaviors was prevalent; people had high motivation to follow the recommended anti-pandemic behaviors, such as limiting time spent outside the home, wearing masks, and maintaining hand hygiene, among others (e.g., Armitage et al., 2021; Czeisler et al., 2021). However, the disruption caused by the pandemic and related policies may have led to non-adherence to COVID-19 protocols and triggered adverse health consequences (Kajiita and Kang’ethe, 2021). To satisfy unmet real-life needs during the pandemic, people may want to go outside to work or explore development opportunities, reduce social distance, and increase social contact.

Thus, a natural hypothesis arises that need dissatisfaction during the pandemic may be related to less support for anti-pandemic behaviors and less compliance with these behaviors. Nese et al. (2022) studied psychological needs and found that dissatisfaction with outdoor sports needs was associated with a reduction in compliance with anti-pandemic behaviors. Vermote et al. (2022) claimed that in times of insecurity (e.g., during a pandemic), the need frustration represents a risk factor for maladjustment. Although there is no direct evidence of the relationship between real-life need dissatisfaction and support for anti-pandemic behaviors, based on the relevant research above, we hypothesized the following:

*H1:* Need dissatisfaction is negatively related to support for anti-pandemic behaviors.

### The multiple mediation of attribution and local government satisfaction

Regarding the mechanism between need dissatisfaction and support for anti-pandemic behaviors, we proposed two pathways. One pathway originates from an individual perspective, according to the health belief model, which posits that individual emotion and risk perception affect health-related behaviors (Rosenstock, 1974, 1977; Weinstein and Nicolich, 1993). Many studies supported the individual emotional and cognitive approach in explaining anti-pandemic behaviors (Kowalski and Black, 2021; Shiloh et al., 2021; Shibani et al., 2022; Anderson and Hobolt, 2023).

The other pathway comes from a social perspective, according to the social identity theory, which posits that the shared identity predicts adherence to protective norms during the pandemic (Tajfel, 1972; Turner, 1982; Shahrabani et al., 2019; Häusser et al., 2020; van Bavel et al., 2022). This inference has been extensively theorized; yet remains largely under-investigated (Stevenson et al., 2021). Researchers have mainly focused on the role of trust when adopting a social perspective since shared identity increased ingroup trust, and they found a positive relationship between trust and compliance behaviors (Rudert et al., 2021; Shi et al., 2021; Ridenhour et al., 2022). The interdependence theory divided trust into interpersonal trust and institutional trust (Kelley and Thibaut, 1978). Between the two, institutional trust belongs to the social perspective. However, results on institutional trust and compliance behaviors varied (see Rudert et al., 2021 for a review). Leong et al. (2022) found that for collectivistic individuals in Slovakia, perceived social norms could explain compliance with public health interventions, but institutional trust could not. Caplanova et al. (2021) also found that institutional trust did not have a statistically significant effect on compliance with face-covering measures.

We argue that local government satisfaction may be a better predictor of compliance behaviors. First, trust in and satisfaction with government are two distinct but related dispositions toward governments associated with social identity (Garritzmann et al., 2021). According to the social identity theory, people tend to evaluate the ingroup more positively than the outgroup; thus they trust and are more satisfied with the ingroup as a whole and the ingroup members (Tajfel and Turner, 1979; Turner, 1982). However, government satisfaction, which is broadly defined as the extent to which government performance and political outcomes meet citizens’ expectations (Warren, 1999; Bailard, 2014), is thought to be a more direct and objective indicator of good governance compared to other more abstract constructs (Bouckaert and van de Walle, 2003). Government satisfaction has been treated as an important indicator of effective governance in China since 1998, when the Citizens Evaluate Government project was launched to survey the public’s satisfaction with local governments (Zhang and Guo, 2021). During the COVID-19 pandemic, the government performance affected how fast the virus would spread, how many people would be infected, and how much normal life would be interrupted. When individuals feel unsatisfied with the government performance, they may tend to not follow the government’s instruction.

Second, the public’s attitude toward central and local government might vary (Saich, 2015). During the COVID-19 pandemic, although the central government sets the core epidemic prevention and control policy, local governments may change and implement it according to the local situation. Moreover, citizens experience more direct interaction with their local governments, and therefore, the satisfaction with the local government may have larger influence on their support for anti-pandemic behaviors.

Nonetheless, researchers seemed to ignore the importance of social evaluation such as local government satisfaction during the COVID-19 pandemic. Indeed, we did not find any empirical studies about local government satisfaction and compliance behaviors; yet, the psychological reactance theory suggests that devaluation of policymakers may lead to non-compliance (Brehm, 1966; Kavvouris et al., 2020; Zhang, 2020; Díaz and Cova, 2022). Additionally, our work in press showed that only satisfaction had a significant indirect effect on recommended behavior. It seems that the individual pathway (need satisfaction – negative emotion/risk perception – compliance) is ineffective, and using exaggeration to trigger negative emotions and risk perception may be useless in promoting compliance with recommended behaviors. However, the social pathway (need satisfaction—local government satisfaction—compliance) may still include an individual mechanism through the mediating role of internal attribution.

According to the attribution theory, people tend to explore reasons for a crisis or an event as naïve psychologists, who try to explain the phenomenon around them without concrete evidence (Heider, 1958; Weiner, 1986, 2010). The attribution of responsibility principally depends on three dimensions (Weiner, 1985, 2014). One of the dimensions adopts an individual versus social perspective differentiates causal elements in terms of their internality or externality, that is, whether an event is caused by something internal or external.

In Heider’s (1958) “naïve analysis of action” model, attribution of causality is influenced by subjective needs and wishes, as well as by more objective evidence. The literature on attribution of success and failure labels the effects of needs and wishes on attribution as defensive, egocentric, egotistic, or self-serving (Zuckerman, 1979). Specifically, it was suggested that people attempt to enhance or protect their self-esteem by taking credit for success and denying responsibility for their failure. This finding is supported by many studies (see Goldberg, 1976 for a review). Since need dissatisfaction could also be considered a type of failure that may reduce self-esteem, higher need dissatisfaction may relate to more external attribution.

Hareli and Weiner (2002) also claimed that in a crisis event, people tend to show anger-related emotions toward responsible actors if they think these have mishandled it. For example, Chung and Lee (2021) found that people feel more anger when they perceive that agents responsible for a situation could have controlled it better. Lee et al. (2022) found that, compared to blaming foreign countries for COVID-19, blaming the federal government was positively related to more hostility toward the federal government. Therefore, people who tend to attribute need dissatisfaction externally may blame the social system more, resulting in lower local government satisfaction; whereas people who tend to attribute need dissatisfaction internally may blame the social system less, resulting in higher local government satisfaction. Consequently, we proposed a multiple mediation. Based on the extent to which the social system was blamed, the H2a involves a social perspective, the H2b involves an individual perspective:

*H2a:* Need dissatisfaction → external attribution →local government satisfaction → support for anti-pandemic behaviors.

*H2b:* Need dissatisfaction → internal attribution →local government satisfaction → support for anti-pandemic behaviors.

Additionally, *via* the role of local government satisfaction, attribution may affect compliance behaviors directly. For example, Weiner (1972a,b, 1974) and Weiner et al. (1971) suggested that individuals’ beliefs about the causes of success and failure may be of major importance in understanding achievement-related behavior. Many studies have proven the role of attribution in achievement-related tasks (Bar-Tal, 1978; Brun et al., 2021). Some studies have also found that attribution mediates the relationship between negative events, such as COVID-19, and emotions (e.g., Estey, 2016; Lee et al., 2022; Olivos et al., 2022), attitudes, (e.g., Claudy et al., 2022; Daryanto et al., 2022) and behaviors (e.g., Zagefka, 2021; Claudy et al., 2022; Kasper et al., 2022). Therefore, we propose another hypothesis:

*H3:* Attribution mediates the relationship between need satisfaction and support for anti-pandemic behaviors.

### The moderation of social class

Since the COVID-19 pandemic increased world inequality (Sidik, 2022), this study also aimed to determine whose needs were less satisfied and the consequences of it. Although pandemic prevention experts referred to people over the age of 65 and/or with comorbidities as vulnerable groups during the pandemic, other groups may also be vulnerable due to unmet needs and other difficulties, such as homelessness (Bodenmann et al., 2020). Some studies have found that compared to their counterparts, people of younger age, lower income, lower educational level, or without a full-time job were less compliant with anti-pandemic behaviors (Armitage et al., 2021; Kowalski and Black, 2021; Kanazawa, 2022; Shibani et al., 2022). According to our previous hypothesis H1, this may be because their normal life was disrupted more, and their needs were more dissatisfied than those of their counterparts. Therefore, we hypothesized:

*H4:* People from lower classes in China have higher need dissatisfaction and less support for anti-pandemic behaviors.

Moreover, there may be social class differences in the attribution of blame. According to Epictetus (a Greek philosopher), the uneducated person blames the world for his misfortunes, the semi-educated person blames himself, and the fully educated blames no one, that there will be a curvilinear relationship between social class, in terms of level of education, and degree of external attribution. In other words, the lower classes will be external in their attributions, the middle classes will tend to be internal, and the upper classes will be external, at least when the outcome is undesirable.

However, Goldberg (1976) reviewed the literature on the naïve perception of causality and summarized that cultures that are more now-oriented (e.g., Spanish cultures) may be less concerned with the prediction and control of future events than cultures that tend to be future-oriented (e.g., Anglo-Saxon cultures). On that basis, one would expect the former to be less external in their attribution than the latter. Since many studies have found that the lower classes are more now-oriented than future-oriented (Griskevicius et al., 2011; Haushofer and Fehr, 2014; Payne, 2017), according to Goldberg’s supposition of cultural differences (1976), the lower classes may tend to attribute less externally when needs are less satisfied.

Therefore, at least two opposing forces affect the relationship between need satisfaction and attribution, which may or may not result in social class differences in causal attribution. Thus, we propose the following open hypothesis:

*H5:* Social classes moderate the relationship between need dissatisfaction and attribution.

### The present study

**Figure 1:**
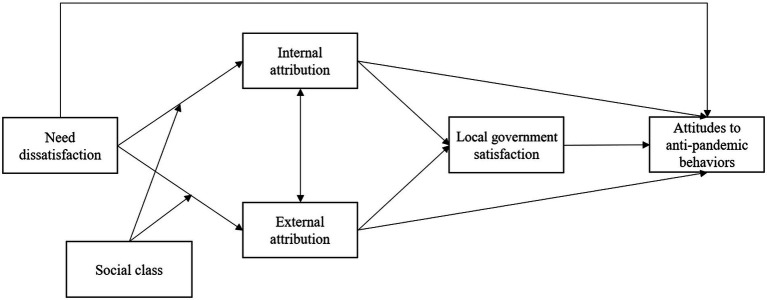
Conceptual model of the study.

The conceptual model of this study is shown in Figure 1. Using this model, the study connected the individual and social perspectives *via* internal and external attribution and contributed to understanding factors that might affect protective behaviors during a global public health event. There are many studies about compliance behaviors during the COVID-19 pandemic; our study adds to the literature in several ways. First, the study was conducted when the Omicron variant, which is more transmissible but less severe than previous variants, became the most prevalent variant worldwide. Considering these characteristics of the Omicron variant, some countries loosened anti-pandemic control and policies (Gollwitzer et al., 2020) and some citizens’ behaviors changed as well (Milani, 2021). Under such conditions, citizens’ attitudes and compliance with anti-pandemic behaviors may be different than those in previous studies. Second, during this period, after over 2 years of COVID-19, needs were largely unsatisfied for many people and the public were in dire need of returning to a normal life. Yet, the contradiction between need dissatisfaction and compliance with anti-pandemic behaviors did not receive enough attention and lacked research. Third, previous studies mainly adopted an individual perspective, exploring the factors affecting compliance behaviors at the individual level, such as personality, risk perception, and emotion, but neglected a social perspective, excluding factors such as performance, evaluation, and satisfaction with the government during the COVID-19.

## Materials and methods

### Recruitment of participants

The data used in the study came from a nationwide series of Chinese Social Mentality Survey (CSMS) executed by the Center of Social Psychology, Institute of Sociology, Chinese Academy of Social Sciences.^1^ We used the data collected in the latest survey round at the time of writing, which focused on the social mentality during the COVID-19 pandemic, from 21 April to 6 May 2022, when participants were recruited through a large online survey platform in China because of the epidemic’s restrictions on offline investigations. Participants came from 29 provinces in Mainland China. Participants from Tibet and Xinjiang were not sampled because of the relatively small populations of the two provinces. To ensure representativeness of the sample, number of participants recruited was limited at the municipal level. For cities with a large COVID-19 spread, such as Shanghai, Beijing, Xi’an, and Wuhan, the quota of recruited participants was 300; for other principal cities that did not have as large of a COVID-19 spread but had high risk due to high mobility, the quota of recruited participants was 100. In the questionnaires, trap and logic test questions were set to ensure data quality. After eliminating abnormal responses and missing data, 6,022 questionnaires were obtained. The participants were aged between 18 and 70 years (*M* = 32.27; *SD* = 8.74), including 2,816 men (46.76%). The characteristics of the sample are listed in Table 1.

**Table 1:** Sample characteristics (*N* = 6,022).

Variable	Mean	SD	Min	Max
Age	32.27	8.74	18	70
Men	0.47	0.50	0	1
Have family members/relatives/friends been infected	0.07	0.26	0	1
Have at least one family member aged 60 years old	0.58	0.49	0	1
Have at least one child aged below 12 years old in the family	0.58	0.49	0	1
Have acquaintances been infected	0.14	0.35	0	1
Have been quarantined	0.66	0.47	0	1
Secondary contact/indirect contact/infected	0.13	0.34	0	1
Monthly household income (nine categories)	5.31	1.98	1	9
Education years	15.67	1.63	6	19
Present employment status-no work	0.05	0.21	0	1
Present employment status-full-time student	0.11	0.32	0	1
Present employment status-at work	0.84	0.37	0	1
Subjective social class	4.90	1.79	1	10

### Measures

#### Independent variable: Need dissatisfaction

In the CSMS survey, need dissatisfaction was measured by asking participants the likelihood of four kinds of difficulties this year: income reduction, business problems, employment problems, and personal development problems. These four indicators met our goals to measure real-life need dissatisfaction since people at all income levels lost money (Sidik, 2022); enterprises were in difficulties, especially small and micro-sized ones that account for a majority of domestic jobs, so when they shut down, many jobs are lost (China Daily, 2022b). And needs like self-achievement will not be met when job and employment needs are unsatisfied. Participants selected ratings from 1 (not at all) to 5 (largely), *а* = 0.879. Participants could also choose “not suitable” for these items if they felt they had no need for income/business/employment/personal development. “Not suitable” was treated as missing data. A higher score indicates higher dissatisfaction with needs.

#### Dependent variable: Support for anti-pandemic behaviors

In the CSMS survey, support for recommended anti-pandemic behaviors was measured by asking participants, “To cope with COVID-19, how necessary do you think the behavior is?” Items were rated from 1 (very unnecessary) to 5 (very much necessary). Behaviors included six aspects recommended by the WHO and Chinese government, including wearing masks indoors, wearing masks outdoors, and washing hands frequently, *а* = 0.867.

#### Mediating variables: Attribution and local government satisfaction

Attribution was measured immediately after questions about need dissatisfaction in the CSMS survey by asking participants, “The reason for the above difficulties lies in?” Five reasons were provided: I did not try hard enough; I lacked ability and talent; COVID-19; pandemic control or quarantine; and poor social governance. The former two reasons represented internal attribution, whereas the latter three represented external attribution. Participants’ ratings ranged from 1 (totally disagree) to 5 (totally agree), *а* = 0.588. The not high Cronbach’s *а* was because the results varied, especially for external attribution.

Local government satisfaction was measured by asking how satisfied participants were with their local government’s performance in 10 items about pandemic prevention and control, such as guaranteeing daily necessities, taking care of seniors and children, and emergency management, and seven items about guaranteeing people’s quality of life, such as employment promotion, public services, and helping the disadvantaged. Participants’ ratings ranged from 1 (not at all) to 5 (very much), *а* = 0.947.

#### Moderating variables: Objective and subjective social class

Social classes can be divided into objective and subjective social classes. Since results of them were often inconsistent, both of them were measured. Compared to objective social class, evidence suggests that subjective social class may be an more robust predictor of various psychosocial outcomes, particularly for certain populations, and may be malleable to change, contrary to the well-documented stability of objective social class across the life course (e.g., Singh-Manoux et al., 2003; McLaughlin et al., 2012; Russell and Odgers, 2020).

We took three objective social class indicators that have been frequently used by scholars, namely, education year, employment status, and monthly household income (e.g., Bourdieu, 1987; Diemer et al., 2013; Highlander and Jones, 2022). Subjective social class was measured using the MacArthur Scale developed by Adler et al. (2000), in which participants are shown a picture of a ladder and told that the social class of people is different; there are people at the top of the ladder and people at the bottom. They are asked in which layer they think they fit. Participants responded with a number from 1 (bottom) to 10 (top).

#### Covariates

Covariates included age, sex, and variables related to the pandemic, such as the present province (to control for the impact of provincial confirmed cases), whether there were any children under 12 years old or seniors over 60 years old in the family (as children and seniors were considered to be vulnerable to infection), and whether family members/relatives/friends had been infected.

## Results

### Preliminary analysis of key variables

The results (Table 2) showed that the needs of the participants were not very satisfied: the average score of need dissatisfaction was 3.27 (*SD* = 0.98), higher than the mid-point of the scale (=3). The most dissatisfied need was income, followed by personal development, employment, and business, indicating that there has been a lasting COVID-19 effect on basic and developmental needs. However, the participants considered anti-pandemic behaviors to be necessary. The average score of support for anti-pandemic behaviors was 4.45, *SD* = 0.63, which was higher than the mid-point of the scale (=3). Participants considered the most necessary behavior was wearing masks outdoors (*M* = 4.58, *SD* = 0.78), while the least necessary behavior was traveling less (*M* = 4.21, *SD* = 0.87).

**Table 2:** Preliminary analysis of key variables (*N* = 6,022).

Variables	M	SD	1	2	3	4	5
1 Need dissatisfaction	3.27	0.98	1				
2 Support for anti-pandemic behaviors	4.45	0.63	−0.06^***^	1.00			
3 Internal attributions	2.92	0.92	0.25^***^	−0.06^***^	1.00		
4 External attributions	3.35	0.68	0.41^***^	0.00	0.18^***^	1.00	
5 Local government satisfaction	3.73	0.70	−0.22^***^	0.27^***^	0.03	−0.25^***^	1.00

^*^p < 0.05; ^**^p < 0.01; ^***^p < 0.001.

Participants tended to attribute need dissatisfaction to external sources (*M* = 3.35, *SD* = 0.68) more than internal sources (*M* = 2.92, *SD* = 0.92). The participants attributed need dissatisfaction to COVID-19 the most, then pandemic control or quarantine, lack of hard work, lack of ability, and poor social governance. This indicates that participants were satisfied with social governance, which is consistent with the results of local government satisfaction, where the average score of local government satisfaction was 3.73 (*SD* = 0.70), higher than the mid-point of the scale (=3). The most satisfactory aspect of pandemic prevention and control was volunteer service, while the least satisfactory was psychological service. The most satisfactory aspect of regular governance that guaranteed people’s quality of life was daily necessities supply, while the least satisfactory was employment promotion.

Correlation analysis showed that the relationship between the independent and dependent variables was not strong (Table 2), suggesting that support for anti-pandemic behaviors may be related with need dissatisfaction mainly through mediating variables. Noteworthily, the external attribution had no significant correlation with support for anti-pandemic behaviors, and the correlation between internal attribution and local government satisfaction was insignificant. These relationships will be later explored in the mediation analysis, when covariates were controlled.

### Testing for the direct associations

When covariates were controlled for, need dissatisfaction negatively predicted support for anti-pandemic behaviors (*B* = −0.048, *p* < 0.001, 95% CI = [−0.064, −0.031]). Thus, Hypothesis 1 was supported.

### The mediation role of attribution

Structural Equation Models were performed by the maximum likelihood method, using Stata 16.1. Considering the large size of the sample, models were fit with the robust variance–covariance matrix. As shown in Model 1 of Table 3, need dissatisfaction positively predicted both internal attribution (*β* = 0.252, *p* < 0.001, 95% CI = [0.225, 0.279]) and external attribution (*β* = 0.412, *p* < 0.001, 95% CI = [0.388, 0.436]). However, the standard coefficient of external attribution was larger than that of internal attribution, indicating that need dissatisfaction has a larger prediction on external attribution than on internal attribution.

**Table 3:** Path coefficients.

Paths	Model 1				Model 2			
	*β*	Value of *p*	95% CILL	95% CIUL	*β*	Value of *p*	95% CILL	95% CIUL
Residual direct effects								
Need dissatisfaction → support	−0.073	0.000	−0.101	−0.046	−0.027	0.052	−0.053	0.000
Direct effects								
Need dissatisfaction → internal attribution	0.252	0.000	0.225	0.279	0.252	0.000	0.225	0.279
Need dissatisfaction → external attribution	0.412	0.000	0.388	0.436	0.412	0.000	0.388	0.436
Internal attribution → support	−0.066	0.000	−0.092	−0.040	−0.092	0.000	−0.116	−0.068
External attribution → support	0.033	0.028	0.004	0.063	0.091	0.000	0.062	0.119
Internal attribution → local government satisfaction					0.072	0.000	0.044	0.100
External attribution → local government satisfaction					−0.261	0.000	−0.290	−0.232
Local government satisfaction → support					0.325	0.000	0.296	0.355
Indirect effects								
Need dissatisfaction → internal attribution → support	−0.017	0.000	−0.023	−0.010	−0.023	0.000	−0.030	−0.017
Need dissatisfaction → external attribution → support	0.014	0.029	0.001	0.026	0.037	0.000	0.025	0.049
Need dissatisfaction → internal attribution → local government satisfaction → support					0.006	0.000	0.004	0.008
Need dissatisfaction → external attribution → local government satisfaction → support					−0.035	0.000	−0.041	−0.029
Total indirect effects								
Need dissatisfaction → internal attribution → support	−0.017	0.000	−0.023	−0.010	−0.017	0.000	−0.024	−0.011
Need dissatisfaction → external attribution → support	0.014	0.029	0.001	0.026	0.002	0.709	−0.010	0.015

Internal attribution had a negative relationship with support for anti-pandemic behaviors (*β* = −0.066, *p* < 0.001, 95% CI = [−0.092, −0.040]), while external attribution had a positive relationship with support for anti-pandemic behaviors (*β* = 0.033, *p* = 0.028, 95% CI = [0.004, 0.063]). The indirect effect of internal attribution on support for anti-pandemic behaviors was-0.017, *p* < 0.001, 95% CI = [−0.023, −0.010], accounting for 16.3% of the total effect. The indirect effect of external attribution on support for anti-pandemic behaviors was 0.014, *p* = 0.029, 95% CI = [0.001, 0.026], accounting for 13.5% of the total effect.

Thus, Hypothesis 3 was supported. However, these two indirect effects on support for anti-pandemic behaviors suppressed each other. That is, internal and external attributions had opposite indirect effects on the relationship between need dissatisfaction and support for anti-pandemic behaviors, resulting in a remaining significant residual direct effect (*β* = −0.073, *p* < 0.001, 95% CI = [−0.101, −0.046]).

### The multiple mediation model of attribution and local government satisfaction

**Figure 2:**
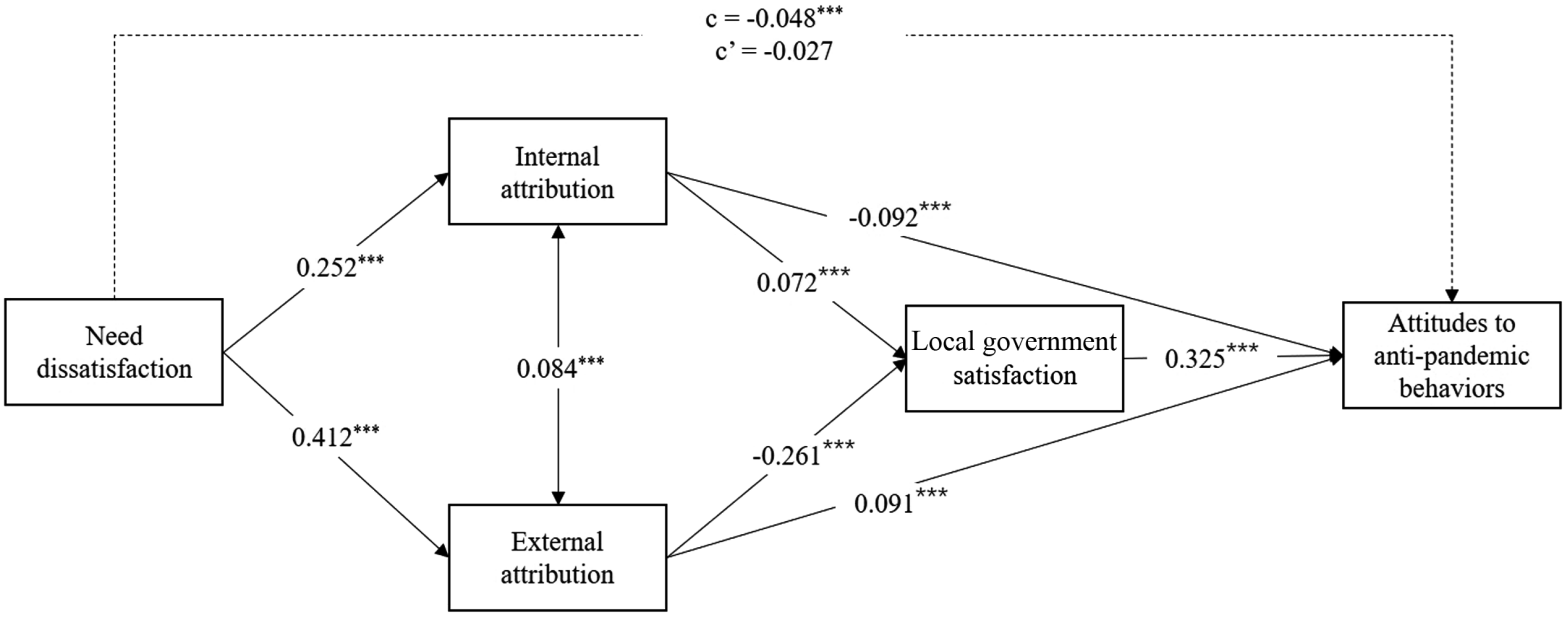
Mediating results.

In Model 2, Table 3, and Figure 2, local government satisfaction was added as the mediator between attribution and support for anti-pandemic behaviors, and the results showed that internal attribution positively predicted local government satisfaction (*β* = 0.072, *p* < 0.001, 95% CI = [0.044, 0.100]), while external attribution negatively predicted local government satisfaction (*β* = −0.261, *p* < 0.001, 95% CI = [−0.290, −0.232]). This means that the effects of internal and external attributions on local government satisfaction suppressed each other.

Moreover, local government satisfaction had a positive relationship with support for anti-pandemic behaviors (*β* = 0.324, *p* < 0.001, 95% CI = [0.296, 0.355]). Therefore, the indirect effects of internal attribution (*β* = −0.023, *p* < 0.001, 95% CI = [−0.030, −0.017]) and external attribution (*β* = 0.037, *p* < 0.001, 95% CI = [0.025, 0.049]) suppressed each other in the relationship between need dissatisfaction and support for anti-pandemic behaviors. In addition, the indirect effects of internal attribution *via* local government satisfaction (*β* = 0.006, *p* < 0.001, 95% CI = [0.004, 0.008]) and external attribution *via* local government satisfaction (*β* = −0.035, *p* < 0.001, 95% CI = [−0.041, −0.029]) also suppressed each other in the relationship between need dissatisfaction and support for anti-pandemic behaviors.

Since the indirect effect of single internal attribution was larger than those of internal attribution and local government satisfaction, the total indirect effect of internal attribution was-0.017, *p* < 0.001, 95% CI = (−0.024, −0.011), accounting for 41.3% of the total effect. The indirect effect of single external attribution and the indirect effect of external attribution and local government satisfaction suppressed each other, and the total indirect effect of external attribution was insignificant (*β* = 0.002, *p* = 0.709, 95% CI = [−0.010, 0.015]). In addition, the residual direct effect became insignificant (*β* = −0.027, *p* = 0.052, 95% CI = [−0.053, 0.000]). Therefore, the relationship between need dissatisfaction was completely mediated by internal attribution and local government satisfaction. Hypothesis 2 was supported.

### Social class differences in need dissatisfaction and support for anti-pandemic behaviors

We performed a linear regression to analyze group differences in need dissatisfaction and support for anti-pandemic behaviors. Age, household income, and subjective social class were treated as a quadratic function, where age was centered and then divided by 10 to better explain the implications of the results. As shown in Table 4, when covariates were controlled, participants who were unemployed at that time reported higher need dissatisfaction than full-time students (*B* = −0.319, *p* < 0.001) and participants who were employed (*B* = −0.163, *p* = 0.013). Subjective social class had a significant quadratic relationship with need dissatisfaction (subjective social class: *B* = −0.261, *p* < 0.001; square of subjective social class: *B* = 0.017, *p* < 0.001). Thus, the relationship between subjective social class and need dissatisfaction was an inverted U-shaped curve, with participants of the middle subjective social class having the highest need dissatisfaction. Thus, Hypothesis 4 was partially supported.

**Table 4:** Linear regression results on need dissatisfaction and support for anti-pandemic behaviors (*N* = 6,022).

Independent variables	Reference variables	Need satisfaction		Support	
		*B*	Value of *p*	*B*	Value of *p*
Constant		4.249	0.000	4.446	0.000
Centered age/10		−0.096	0.000	−0.010	0.509
Square of centered age/10		−0.025	0.049	−0.020	0.075
Men	Women	−0.002	0.921	−0.092	0.000
Have family members/relatives/friends been infected	No	0.118	0.035	−0.231	0.000
Have at least one family member aged 60 years old	No	0.127	0.000	0.050	0.002
Have at least one child aged below 12 years old in the family	No	0.059	0.030	0.012	0.535
Have acquaintances been infected	No	0.101	0.010	0.058	0.024
Have been quarantined	No	0.179	0.000	0.033	0.063
Secondary contact/indirect contact/infected	No	0.106	0.009	−0.132	0.000
Monthly household income (nine categories)		−0.015	0.678	−0.046	0.083
Square of monthly household income		0.000	0.972	0.003	0.146
Education year		−0.015	0.069	−0.004	0.530
Present employment status-full-time student	Present employment status-no work	−0.319	0.000	0.106	0.050
Present employment status-at work	Present employment status-no work	−0.163	0.013	0.062	0.214
Subjective social class		−0.261	0.000	0.093	0.000
Square of subjective social class		0.017	0.000	−0.011	0.000
*R* ^2^		0.086		0.047	
*F*		13.250	0.000	5.180	0.000

Province was controlled.

Support for anti-pandemic behaviors also had a significant quadratic relationship with subjective social class (subjective social class: *B* = 0.093, *p* < 0.001; square of subjective social class: *B* = −0.011, *p* < 0.001); however, the relationship between subjective social class and support for anti-pandemic behaviors was a U-shaped curve, with participants of the middle subjective social class supporting for the anti-pandemic behaviors the least. Other social class variables had no significant relationship with need dissatisfaction or support for anti-pandemic behaviors. Therefore, it seems that participants whose needs were less satisfied considered individual anti-pandemic behaviors more necessary, which was consistent with the findings above.

### The moderation of social class between need dissatisfaction and attribution

**Figure 3:**
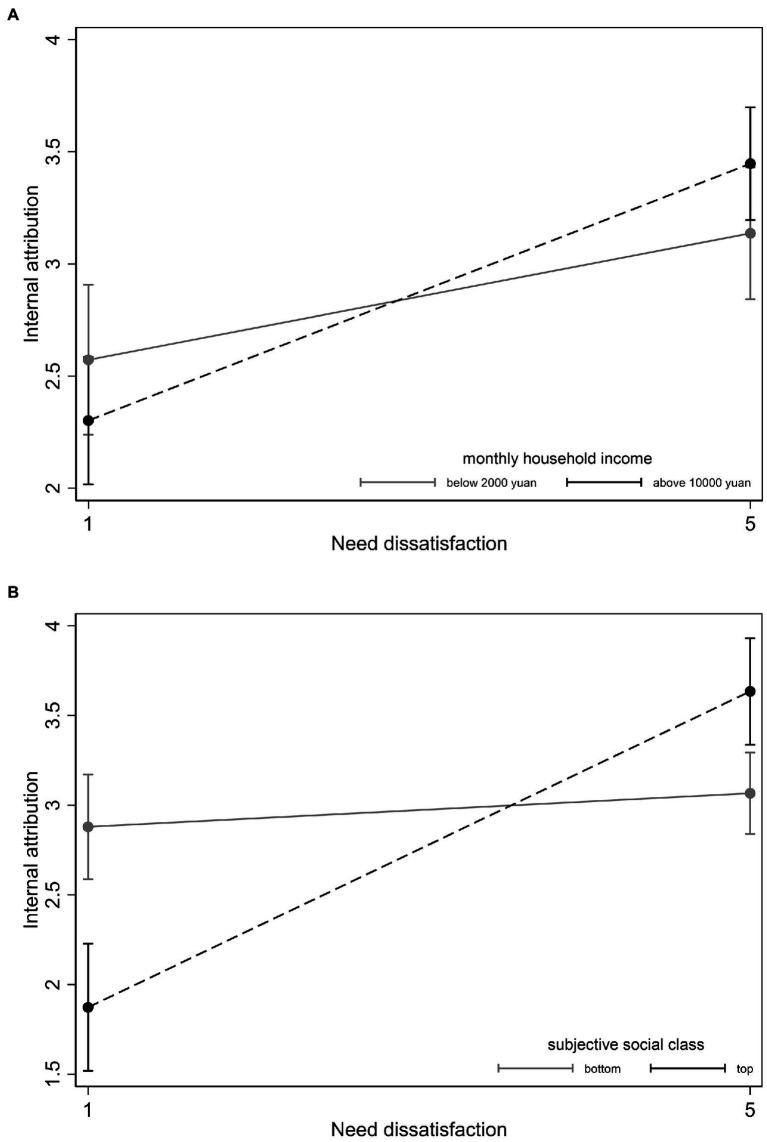
The moderation of household income **(A)** and subjective social class **(B)** between need dissatisfaction and internal attribution.

Table 5 shows the moderation results. Only income (*B* = 0.018, *p* = 0.006, 95% CI = [0.005, 0.031]) and subjective social class (*B* = 0.044, *p* < 0.001, 95% CI = [0.029, 0.058]) played significant moderating roles in the relationship between need dissatisfaction and internal attribution. Specifically, when need dissatisfaction was high, participants with higher income and subjective social class tended to attribute more internally (Figures 3A,B). None of the other objective social class variables had a significant moderating role in the relationship between need dissatisfaction and external attribution; thus, Hypothesis 7 was partially supported.

**Table 5:** Moderation results.

Variable	Reference	Internal attribution				External attribution			
Constant		2.567^***^	1.712^***^	2.405^***^	2.998^***^	2.030^***^	2.188^***^	1.692^***^	2.009^***^
Centered age/10		−0.196^***^	−0.197^***^	−0.197^***^	−0.195^***^	−0.006	−0.006	−0.006	−0.006
Square of centered age/10		0.050^***^	0.052^***^	0.050^***^	0.050^***^	0.006	0.007	0.009	0.007
Men	Women	−0.063^**^	−0.062^**^	−0.062^**^	−0.061^**^	0.002	0.002	0.001	0.002
Have family members/relatives/friends been infected	No	0.099	0.102^*^	0.100	0.091	−0.004	−0.004	−0.003	−0.004
Have at least one family member aged 60 years old	No	0.003	0.001	0.000	0.000	−0.046^**^	−0.046^**^	−0.046^**^	−0.046^**^
Have at least one child aged below 12 years old in the family	No	0.047	0.047	0.047	0.046	0.012	0.012	0.012	0.011
Have acquaintances been infected	No	−0.021	−0.022	−0.023	−0.021	0.034	0.033	0.034	0.033
Have been quarantined	No	0.010	0.011	0.012	0.013	0.081^***^	0.081^***^	0.080^***^	0.082^***^
Secondary contact/indirect contact/infected	No	−0.012	−0.008	−0.010	−0.017	−0.014	−0.014	−0.012	−0.014
Monthly household income (nine categories)		−0.052	0.017	0.019	0.019	0.007	0.030	0.028	0.029
Square of monthly household income		0.000	0.000	−0.001	0.000	−0.003	−0.003	−0.003	−0.003
Education year		−0.016^*^	0.016	−0.017^*^	−0.014	0.018^**^	0.000	0.017^**^	0.018^**^
Full-time student	No work	−0.006	−0.006	−0.209	−0.023	−0.042	−0.043	0.270	−0.044
At work	No work	0.037	0.037	−0.161	0.023	0.047	0.046	0.281	0.045
Subjective social class		0.016	0.022	0.019	−0.155^***^	0.008	0.008	0.010	−0.011
Square of subjective social class		−0.002	−0.003	−0.003	0.000	0.000	0.000	−0.001	0.000
Need dissatisfaction		0.123^**^	0.377^**^	0.169^**^	0.003	0.254^***^	0.203^*^	0.349^***^	0.260^***^
Need dissatisfaction × monthly household income		0.018^**^				0.006			
Need dissatisfaction × education year			−0.010				0.005		
Need dissatisfaction × full-time student	No work			0.057				−0.090	
Need dissatisfaction × at work	No work			0.055				−0.066	
Need dissatisfaction × subjective social class					0.044^***^				0.005

^*^p < 0.05; ^**^p < 0.01; ^***^p < 0.001.

## Discussion

### Main findings

This study found that the participants had low need satisfaction, indicating that the pandemic has greatly affected their lives. Regardless of low need satisfaction, Chinese people still highly agreed with anti-pandemic behaviors. Studies done in other countries also found a high compliance with anti-pandemic behaviors (e.g., Armitage et al., 2021; Czeisler et al., 2021); but some research took a cultural perspective and attributed the high compliance of Chinese people to collectivism. For instance, Lu et al. (2021) found that collectivism predicted mask use during the COVID-19 pandemic. Although collectivism may make Chinese people positive about these behaviors, the study found a negative relationship between need dissatisfaction and support for anti-pandemic behaviors, which was completely mediated by attribution and local government satisfaction.

However, the mediating role of internal/external attribution and local government satisfaction sometimes suppressed each other. Internal and external attribution act as double-edged swords. First, *internal* attribution *negatively* related with support for anti-pandemic behaviors, while *external* attribution *positively* related with support for anti-pandemic behaviors. That is, if participants attributed need dissatisfaction to personal inability or inexertion, they tended to disagree with anti-pandemic behaviors. On the contrary, if participants attributed need dissatisfaction to external reasons, such as the COVID-19 pandemic or poor social governance, they tended to support anti-pandemic behaviors. This may be because they expected that by applying stricter anti-pandemic behaviors, the pandemic could be controlled more quickly; thus, their needs could be satisfied.

Second, *via* the mediation of local government satisfaction, *internal* attribution became *positively* related with support for anti-pandemic behaviors, while *external* attribution became *negatively* related with support for anti-pandemic behaviors. This is because internal attribution increased local government satisfaction, whereas external attribution decreased it; and higher local government satisfaction predicted more positive support for anti-pandemic behaviors. These results were consistent with the attribution theory, which posits that blaming an external actor will increase the anger as well as de-evaluation of the actor (Heider, 1958; Weiner, 1986, 2010; Hareli and Weiner, 2002).

Nonetheless, the study also found that the indirect effect of single internal attribution was larger than those of internal attribution and local government satisfaction; thus, the total indirect effect of internal attribution was negative and completely mediated the negative relationship between need dissatisfaction and support for anti-pandemic behaviors. Therefore, even though internal attribution is a double-edged sword, attributing need dissatisfaction to personal inability or inexertion may stimulate people to work harder or go outside for more jobs or development opportunities, which, consequently, could lead to less compliance behaviors.

Moreover, the study found that participants tended to attribute need dissatisfaction more to external attributions than internal attributions. This was also consistent with the attribution theory, which posits that people attempt to enhance or protect their self-esteem by taking credit for success and denying responsibility for their failure (Heider, 1958; Goldberg, 1976; Zuckerman, 1979). However, the indirect effect of single external attribution was similar with those of external attribution and local government satisfaction; thus, the total indirect effect of external attribution was insignificant. In other words, even though internal attribution reduced compliance behaviors, increasing external attribution had little effect in promoting compliance.

Therefore, to promote compliance, simply changing people’s attribution may not be helpful; we need to change the mechanism or logic behind these attributions. First, increasing the positive impact of internal attribution and local government satisfaction on support for anti-pandemic behaviors by guiding people who attributed need dissatisfaction to personal reasons to recognize the role of local government in preventing and controlling the pandemic spread, as well as guaranteeing people’s quality of life. Second, increasing the positive impact of external attribution solely on support for anti-pandemic behaviors by guiding people who attributed need dissatisfaction to external reasons to realize that by applying stricter anti-pandemic behaviors, the pandemic could be controlled more quickly; thus, their needs could be satisfied, instead of blaming the government.

As New York’s Governor, Andrew Cuomo said, “It’s not about me, it’s about we” (Jetten et al., 2020). We need to create a “we” term between the public and the local government, promote a positive interaction between them, and bring people together psychologically to stay apart physically.

In addition, this study found that participants who were unemployed at the time of the study reported higher need dissatisfaction than full-time students and participants who were employed. The COVID-19 pandemic has caused a massive increase in unemployment. For example, since the outbreak of Omicron variant in April 2022, the Chinese employment market prosperity index, computed by market recruitment demand, showed that market job applications have fallen to 1.35 from 1.56 in the first quarter of 2022. The impact on social status, self-esteem, and fear of losing future income can have a greater impact than financial damage itself (Detrixhe, 2020). For emotional well-being, employment is typically more effective than an unemployment check (Detrixhe, 2020). Therefore, the promotion of economic growth and employment rates has become increasingly important in the context of the COVID-19 pandemic and global economic slowdown.

Moreover, subjective social class had an inverted U-shaped relationship with need dissatisfaction, with participants belonging to the subjective middle social class having the highest need dissatisfaction, reflecting a type of middle-class anxiety. Many media reports have shown that the middle class in China is anxious about housing prices, overwork, and raising children, and middle-class anxiety is a real phenomenon (Mattimore, 2012). Our findings indicate subjective dissatisfaction among the middle class. Many previous studies have treated variables such as income or subjective social class as linearly dependent, but a quadratic or even cubic function model should also be considered.

This study also found a moderating effect of social class on the relationship between need dissatisfaction and internal attribution. Specifically, when needs were not met, participants with higher income and subjective social class tended to attribute more internally. This finding supported Epictetus, but refuted the now-oriented or future-oriented supposition (Goldberg, 1976). It alerted us that even though people of lower social class had more need dissatisfaction and less support for anti-pandemic behaviors so far, once the needs of higher social class were heavily unsatisfied, it may result in much less compliance of behaviors than that of present.

### Implications

This study examined the need-attribution-behaviors model in the attribution theory (Heider, 1958; Weiner, 1986, 2010) and social identity theory (Tajfel, 1972; Turner, 1982; Shahrabani et al., 2019; Häusser et al., 2020; van Bavel et al., 2022) of the COVID-19 pandemic, which could be helpful in understanding individuals’ attitudes and behaviors in the context of major global public health events. Moreover, the study found a double-edged role for internal and external attributions, which has rarely been mentioned in previous studies. The study also found that internal and external attributions may have two separate mechanisms, which provides direction for future research.

This study showed that there should be a focus on the need satisfaction of people from the lower and middle classes. Promoting economic growth and employment rates should be a top priority for all countries. In addition, to increase compliance, we need to create a “we” term between the public and the local government and promote a positive interaction between the two parties. There are two ways to do this: first, by making people aware that the current situation requires more cooperation and compliance to overcome the pandemic and to return to normal life as soon as possible; second, by increasing government performance to increase citizens’ satisfaction and support for anti-pandemic behaviors.

### Limitations

This study has several limitations. First, it only measured the locus of causality of attribution. In achievement-related research, internal or external attribution of failure alone cannot predict achievement-related behaviors; other attribution dimensions, such as stability, should be included (Bar-Tal, 1978; Zuckerman, 1979; Brun et al., 2021). This may be the same in pandemic-related research, so future research could consider more dimensions of attribution and explore their interaction with anti-pandemic attitudes and behaviors.

Second, need dissatisfaction is related with relative deprivation (Smith et al., 2012), since both involve a feeling of deprivation; however, relative deprivation involves comparison with others (Smith et al., 2012), which need dissatisfaction does not. In other words, this study only studied the absolute deprivation, that is, need dissatisfaction; but further research could introduce the theory of comparison and relative deprivation, exploring the relationship between relative deprivation and compliance with anti-pandemic behaviors.

Third, the applicability of the results in other countries or cultures should be investigated. For example, need dissatisfaction was negatively related with support for anti-pandemic behaviors in China; however, in countries where epidemic prevention and control were loosened, would their citizens blame need dissatisfaction on loosened policies and then support anti-pandemic behaviors? Moreover, since there are many differences in cognition, attitudes, and behaviors between individualistic and collectivistic cultures (Lu et al., 2021; Leong et al., 2022), and now-oriented and future-oriented cultures (Goldberg, 1976), cultural differences in the relationship and mechanism between need dissatisfaction and compliance should be considered and explored in the future studies.

Fourth, other data could be used, other measurements could be introduced and other research designs could be applied to test the repeatability of the study. For example, the study used data from an online survey round of CSMS; once possible, data from an offline survey with random sampling could be used. Furthermore, limited by the survey data and research aim, there were no well-established instruments that could be used; future researchers could develop more mature scales according to the pandemic situation. In addition, the study used self-report data which was easily to be affected by social desirability, once possible, data from other sources, such as implicit measurement could be considered. Finally, this was a cross-sectional study; in the future, a cross-lag method should be used to clarify the mechanisms of variables.

### Conclusion

The study analyzed the need dissatisfaction of Chinese people after over 2 years of the COVID-19 pandemic and its consequences on their support for anti-pandemic behaviors. The participants reported high need dissatisfaction and support for anti-pandemic behaviors; yet, need dissatisfaction was negatively related to support for anti-pandemic behaviors and was completely mediated by attribution and local government satisfaction. Internal and external attribution acted as a double-edged sword: they negatively/positively related with support for anti-pandemic behaviors, while they became positively/negatively related *via* the mediation of local government satisfaction. People from lower social classes, especially those who are unemployed and from the subjectively middle class, reported higher need dissatisfaction and less support for anti-pandemic behaviors compared to their counterparts. Social class moderated the relationship between need dissatisfaction and internal attribution; when needs were dissatisfied, participants of higher income and subjective social class tended to attribute more internally. Promoting a positive interaction between the public and local governments may be helpful to create a shared identity to prevent and control the pandemic together.

## Data availability statement

The raw data supporting the conclusions of this article will be made available by the authors, without undue reservation.

## Ethics statement

The studies involving human participants were reviewed and approved by Institute of Sociology, Chinese Academy of Social Sciences. Written informed consent for participation was not required for this study in accordance with the national legislation and the institutional requirements.

## Author contributions

YZ contributed to research design, data analyzing, and manuscript writing. JW contributed to survey design, data collecting, and manuscript revising. All authors contributed to the article and approved the submitted version.

## Funding

The study was funded by Key Projects of Philosophy and Social Sciences Research, Ministry of Education (grant number: 21JZD038) and the National Social Science Foundation (grant number: 21CSH045).

## Conflict of interest

The authors declare that the research was conducted in the absence of any commercial or financial relationships that could be construed as a potential conflict of interest.

## Publisher’s note

All claims expressed in this article are solely those of the authors and do not necessarily represent those of their affiliated organizations, or those of the publisher, the editors and the reviewers. Any product that may be evaluated in this article, or claim that may be made by its manufacturer, is not guaranteed or endorsed by the publisher.

## Footnotes

^1^For more information about the concept and structure of social mentality, please see the Yang (2019).
